# Bullous eczema with hyaluronic acid deposition: A case report

**DOI:** 10.1016/j.jdcr.2025.10.007

**Published:** 2025-10-11

**Authors:** Nobushige Kohri, Kazuki Yatsuzuka, Satoshi Yoshida, Katsuhiko Nishihara, Ken Shiraishi, Yasuhiro Fujisawa, Jun Muto

**Affiliations:** Department of Dermatology, Ehime University Graduate School of Medicine, Toon, Ehime, Japan

**Keywords:** bullous eczema, dyshidrotic eczema, hyaluronic acid, subepidermal blister

## Introduction

Bullous eczema is a rare dermatological condition characterized by subepidermal blisters, first described by Atteh et al.^1^ This condition exhibits clinical and histological features that overlap with bullous pemphigoid (BP) but lacks detectable autoantibodies. While the pathogenesis of bullous eczema remains unclear, a previous study indicated that the increased production of hyaluronic acid (HA) by epidermal keratinocytes stimulated by cytokines plays a crucial role in the formation of spongiosis,^2^ considered a critical finding in eczema. In this case report, we confirmed and compared HA deposition in patients with bullous eczema and BP, with reference to the HA deposition we previously reported in dyshidrotic eczema.^3^

## Case report

A 52-year-old male presented with vesicles on the hands and feet and erythema and fluid-filled vesicles on the trunk and extremities. His past medical history included allergic rhinitis with no history of metal allergy, and there was no use of any new medications before the onset of the skin symptoms, and no symptoms suggestive of viral infection. The patient reported noticing itchy red papules on his lower legs 2 months prior to his visit to our department. At that time, no mucosal symptoms were evident. Initial treatment with topical clobetasol propionate showed no improvement. Subsequently, oral prednisolone (10 mg/day) was prescribed, leading to the temporary resolution of the lesions. However, the rash recurred and worsened 2 weeks before his visit to our department.

**Fig 1:**
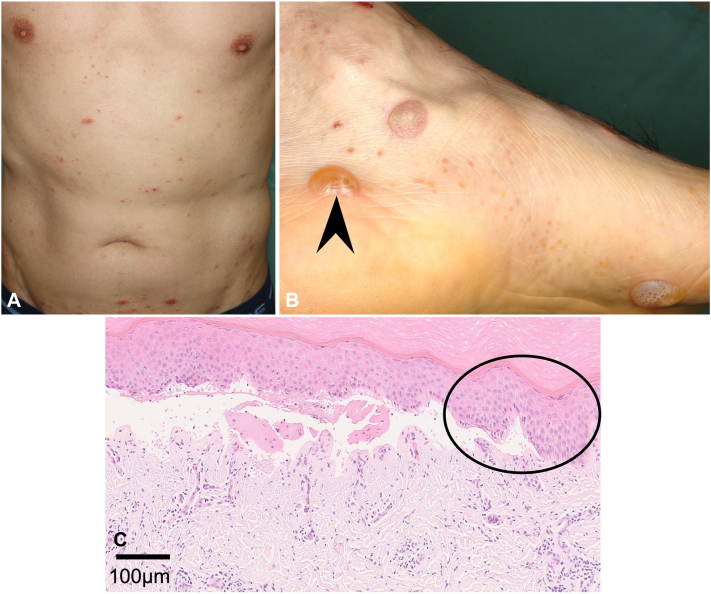
Clinical presentation and histopathological findings at the initial visit. The patient presented with multiple fluid-filled vesicles on the trunk (**A**). On the soles and ankles, deep-seated vesicles and tense bullae displaying tapioca pudding-like patterns were observed (**B**). A skin biopsy from a tense bulla on the ankle (*arrowhead*) revealed acanthosis, spongiosis (*circle*), a subepidermal blister containing red blood cells, and lymphocytic and eosinophilic infiltration around the dermal vessels (**C,** Hematoxylin-eosin stain).

Clinical examination revealed erythema and fluid-filled vesicles on the trunk (Fig 1, *A*) and extremities, along with erosive lesions with exudate on the scalp. Vesicles and tense bullae were observed on the wrists, soles, and ankles (Fig 1, *B*). Notably, the tense bullae on the soles and ankles exhibited a characteristic “tapioca pudding” appearance (Fig 1, *B*), commonly associated with dyshidrotic eczema.^4^

Initial differential diagnoses included dyshidrotic eczema, autosensitization dermatitis, BP, and dyshidrotic eczema-like pemphigoid. Laboratory findings revealed elevated eosinophil counts (8.4%) and IgE levels (661 IU/mL). Serum autoantibody tests for autoimmune bullous disorders were negative. A biopsy from a tense bulla on the ankle (Fig 1, *B*) demonstrated acanthosis, spongiosis, subepidermal blistering, and lymphocytic and eosinophilic infiltration around the dermal vessels (Fig 1, *C*). Direct immunofluorescence of the patient’s perilesional skin was negative for C3, IgG, IgA, and IgM, ruling out autoimmune bullous disorders.

**Fig 2:**
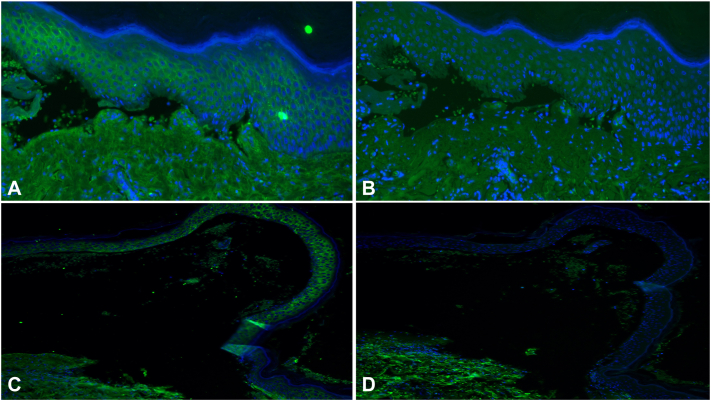
Hyaluronic acid (HA) staining was performed using a previously reported method.^6^ Biopsy specimens from a bullous eczema patient (**A, B**) and a bullous pemphigoid patient (**C, D**) were used. *Blue signals* represent Hoechst staining. *Green signals* between keratinocytes indicate the presence of HA-binding protein (**A, C**). Following treatment with hyaluronidase, HA between the keratinocytes in the blister roof and the surrounding area was degraded (**B, D**). None of the biopsy specimens showed acantholytic keratinocytes covered with HA within the blisters. (**A-D**, HA stain; original magnification: **A, B**; × 200; **C, D**; × 100)

Most lesions were consistent with dyshidrotic eczema and autosensitization dermatitis; however, the presence of subepidermal blisters extending beyond the palms and soles was atypical. Prodromal BP was also considered; however, a review of the literature revealed bullous eczema, which exhibits BP-like clinical features in the absence of autoantibodies. To confirm the eczematous nature of the lesion, HA staining was performed on the biopsy specimen. Strong HAdeposits were observed between keratinocytes around the blister (Fig 2, *A* and *B*), supporting a diagnosis of bullous eczema.

Treatment with topical and oral corticosteroids was continued, and oral minocycline was added as a precaution until BP could be definitively excluded. Prednisolone was increased to 15 mg/day for 2 weeks and tapered as the lesions improved. The patient ultimately achieved complete resolution of the skin lesions.

## Discussion

Atteh et al first described bullous eczema in 2021, reporting 6 cases with BP-like clinical features and histological overlap between eczema and BP, yet lacking detectable autoantibodies.^1^ Unlike dyshidrotic eczema, the bullae in bullous eczema often extend beyond the palms and soles and are generally larger.^1^

In eczematous lesions, interleukin-4, interleukin-13, and interferon-γ reduce E-cadherin expression, promoting HA production and deposition between keratinocytes.^2^ This process contributes to spongiosis and, in rare cases, progression from intraepidermal vesiculation to subepidermal blistering.^5^^,^^6^ Our group previously demonstrated the utility of HA staining in distinguishing dyshidrotic eczema from palmoplantar pustulosis.^3^ In this case, HA staining confirmed the eczematous nature of the lesion, supporting the diagnosis of bullous eczema. Moreover, acantholytic keratinocytes covered with HA within the vesicle, which are often seen in dyshidrotic eczema, were not observed. We additionally performed HA staining on the lesions of a BP patient. Similar to bullous eczema, HA deposition was observed in the epidermis above the blister. Since interleukin-4/13 has been reported to play an essential role in the pathogenesis of BP,^7^ HA deposition was thought to have been observed as a result. Furthermore, keratinocytes covered with HA, as seen in dyshidrotic eczema, were not observed inside the blister. Taken together, the HA deposition patterns in bullous eczema and BP are similar, but distinct from those seen in dyshidrotic eczema. Hence, the pathogenesis of bullous eczema and dyshidrotic eczema may differ significantly. Given the rarity of bullous eczema, accumulating more cases and conducting further research is crucial to clarifying its pathology and developing targeted therapies.

## Declaration of generative AI and AI-assisted technologies in the writing process

Artificial intelligence (Chat GPT) was used for writing assistance.

## Conflicts of interest

None disclosed.
